# The Epidermal Growth Factor Receptor Increases Cytokine Production and Cutaneous Inflammation in Response to Ultraviolet Irradiation

**DOI:** 10.1155/2013/848705

**Published:** 2013-06-25

**Authors:** Taghrid Bahig El-Abaseri, Brianna Hammiller, Susan K. Repertinger, Laura A. Hansen

**Affiliations:** ^1^Department of Biomedical Sciences, Creighton University, Omaha, NE 68178, USA; ^2^Medical Biochemistry Department, School of Medicine, Suez Canal University, Ismailia, Egypt; ^3^Department of Pathology, Creighton University Medical Center, Omaha, NE 68178, USA; ^4^Department of Biomedical Sciences, School of Medicine, Creighton University, 2500 California Plaza, Omaha, NE 68178, USA

## Abstract

The epidermal growth factor receptor (EGFR) is activated in cutaneous keratinocytes upon ultraviolet (UV) exposure and has been implicated in ultraviolet-(UV-)induced inflammation and skin tumorigenesis. *Egfr* mutant mice and EGFR inhibitors were used to investigate the hypothesis that EGFR activation augments inflammation following UV irradiation. Topical treatment of mouse skin with the EGFR inhibitor AG1478 before UV exposure suppressed UV-induced erythema, edema, mast cell infiltration, and neutrophil infiltration. Genetic ablation of *Egfr* and EGFR inhibition by AG1478 also suppressed the increase in the proinflammatory cytokines tumor necrosis factor **α** (TNF-**α**), interleukin-1**α**, KC (murine IL-8), and cyclooxygenase-2 (COX-2) after UV exposure of cultured keratinocytes. Finally, genetic ablation of inhibition of EGFR in cultured keratinocytes decreased p38 activation after UV, while inhibition of p38 kinase reduced COX-2 expression after UV. These data demonstrate that EGFR regulates multiple aspects of UV-induced inflammation and suggest activation of p38 kinase leading to increased COX-2 and cytokine expression as one mechanism through which it acts.

## 1. Introduction

Epidermal growth factor receptor (EGFR) signaling is involved in important aspects of cutaneous biology, including the regulation of epidermal proliferation, apoptosis, cell adhesion, and migration. For example, EGFR signaling appears to be important for such adaptive biologic processes as wound healing [1]. On the other hand, excessive EGFR signaling may participate in processes that are ultimately destructive to skin, such as in the skin's carcinogenic response to ultraviolet (UV) exposure [2–4].

Solar UV radiation is a major environmental hazard that generates reactive oxygen species, induces DNA damage, and leads ultimately to skin inflammation, photoaging, and cancer development [5]. Erythema and edema are the grossly visible signs of UV-induced inflammation in mammalian skin [6]. These changes are associated histologically with dermal infiltration of neutrophils, followed later by macrophages and mast cells [7]. These cellular events are accompanied or preceded by the release of a wide variety of proinflammatory mediators, including certain enzymes and cytokines. For example, following skin exposure to UV light, levels of the pro-inflammatory enzyme cyclooxygenase-2 (COX-2) are increased [8], which in turn leads to production of prostaglandin E2 (PGE2), a potent mediator of UV-induced skin erythema [9]. In addition to the activation of various enzymes in all nucleated cells in the skin, cells secrete several cytokines in response to UV exposure. UV-induced cytokines include interleukin-(IL-)8 [10–12], IL-1*α* [13, 14], and TNF-*α* [12, 15]. Release of cytokines in response to UV plays a central role in the autorecruitment and activation of inflammatory cells [16] as well as the production of matrix metalloproteases (MMPs) [17], contributing to the final pathological changes seen in chronic sun-damaged skin.

Since UV exposure activates EGFR indirectly through a mechanism involving reactive oxygen species inactivation of protein tyrosine phosphatase kappa [18], multiple EGFR-dependent signaling pathways may contribute to the physiological and histological effects seen in UV-irradiated skin. In particular, p38 mitogen-activated protein (MAP) kinase plays a critical role in regulating cellular responses to UV. For example, p38 kinase is activated in cultured keratinocytes [19] and in skin upon UV exposure [3, 19]. p38 kinase upregulates the expression of the pro-inflammatory cytokine IL-8 in keratinocytes following UV exposure [20]. Interestingly, inhibition of p38 kinase decreases UV-induced expression of KC (murine IL-8) [18], COX-2 [18, 21, 22], and PGE2 [21], thus lessening skin erythema [18]. 

While deregulated EGFR signaling in the skin in response to UV irradiation is implicated in epidermal hyperplasia, proliferation, apoptosis, and tumor formation [3, 4], its modulation of the inflammatory response is not fully understood. Therefore, the current study was designed to investigate the role of EGFR signaling in UV-induced skin inflammation. We investigated the role of EGFR in the molecular mechanisms implicated in UVA/B-induced skin inflammation *in vitro* using *Egfr*-null and wild-type keratinocytes and an EGFR inhibitor, and *in vivo* using EGFR inhibitor-treated mouse skin. Our data showed that EGFR led to activation of p38 kinase, increased COX-2 levels, enhanced expression of the pro-inflammatory cytokines, and increased dermal infiltration of neutrophils and mast cells following acute exposure to UV. 

## 2. Methods

### 2.1. Cell Culture

Primary keratinocytes were isolated from newborn CD-1 mouse skin or from *Egfr* ^−/−^ null mice on a CD-1 background, as described previously [23]. In brief, the skin was floated on trypsin (Invitrogen, Carlsbad, CA, USA) at 4°C overnight; the epidermis separated from the dermis; the epidermis was minced, triturated, and centrifuged in SMEM (Invitrogen) containing 8% Chelex-treated (Bio-Rad, Hercules, CA, USA) fetal bovine serum (Gemini Bioproducts, Woodland, CA, USA). Keratinocytes grown to 70–80% confluence were exposed to either 200 or 600 J/m^2^ UVA/B or were sham irradiated in a thin layer of phosphate-buffered saline containing 0.05 mmol/L calcium. Some keratinocytes were pretreated with 1 *μ*mol/L AG1478 or 5 *μ*mol/L PD169316 (Calbiochem, San Diego, CA, USA), dissolved in DMSO or DMSO alone 1 (PD169316) or 2 h (AG1478) before UV exposure, and refed fresh medium containing the inhibitor or vehicle alone immediately after UV exposure. For cytokine analysis, cell lysate suspended in TRAP_EZE_ CHAPS lysis buffer (Chemicon, Temecula, CA, USA) was analyzed using a Luminex instrument (Upstate Biotechnology, Charlottesville, VA, USA) according to the manufacturer's protocol.

### 2.2. Animals

 The dorsal hair of mice was trimmed with electric clippers and a shaver at least one day prior to UV exposure. Some mice were topically treated or injected intraperitoneally (i.p.) with 150 mg/kg of AG1478 in DMSO or the vehicle DMSO alone 2 h prior to UV exposure using a protocol we have previously published [3]. FS40T12 sunlamps (Westinghouse, NJ, USA) emitted approximately 70% UVB, 30% UVA, and 1% UVC, with a total output of 1.46 mW/cm^2^, as measured with radiometric photodetector probes (Oriel, Stratford, CT, USA). Skinfold thickness in age-matched, UV-exposed homozygous female Tg.AC mice on an FVB/N background was measured using calipers. *Egfr*-null and wild-type newborn mice on a CD-1 background were genotyped as described previously [24]. Erythema was assessed as the presence or absence of skin redness twenty-four hours following UV exposure. All animal experiments were performed with the approval and oversight of Creighton University's Institutional Care and Use Committee.

### 2.3. Immunofluorescence and Microscopy

Dorsal skin sections were fixed in formalin and stained with hematoxylin and eosin. Neutrophils were identified and counted in three representative microscopic fields on hematoxylin- and eosin-stained sections with the investigator blinded as to the identity of the samples. Mast cells were identified and similarly counted following immunofluorescence with an antitryptase antibody (Cell Signaling) and DAPI (Vector Labs) to identify the nuclei. 

### 2.4. Immunoblotting

Cells were lysed in buffer containing 10 mM Tris pH 7.4, 150 mM NaCl, 10% glycerol, 1% Triton X-100, 1 mM EDTA, complete protease inhibitor (Roche, Germany), 1 mM Na_3_VO_4_, 1.5 M EGTA, and 10 M NaF. Protein levels were quantitated using the BioRad assay (BioRad, Hercules, CA, USA). Immunoblotting with antibodies recognizing COX-1 (Cayman Chemical, Ann Arbor, MI), COX-2 (Cayman), phospho-p38 kinase (Cell Signaling, Beverly, MA, USA), p38 kinase (Cell Signaling), phospho-ATF2 (Cell Signaling), and actin (Sigma, St.Louis, MO, USA) was performed using the appropriate horseradish peroxidase-conjugated secondary antibody (Cell Signaling) and chemiluminescence reagents (Pierce, Rockford, IL, USA). Densitometry was performed using 1DScan software (Scanalytics, Fairfax, VA, USA).

## 3. Results

### 3.1. Inhibition of EGFR Decreases UV-Induced Erythema and Edema

**Figure 1:**
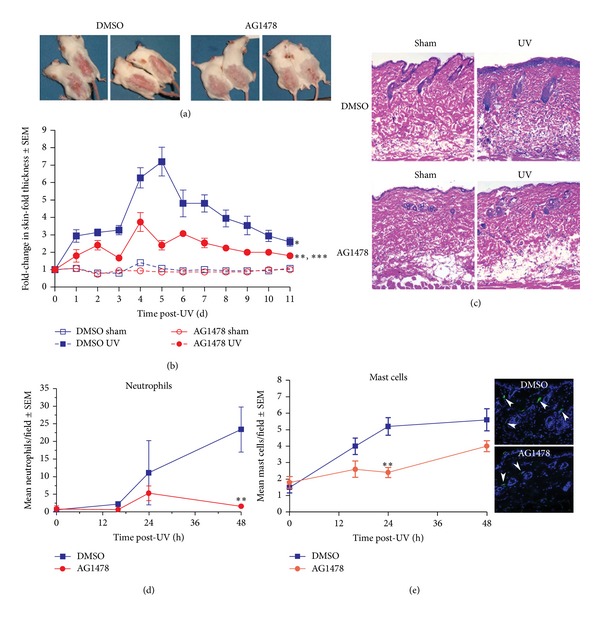
EGFR inhibition results in less acute UV-induced skin injury. Mice were treated topically with AG1478 or vehicle alone and exposed to 10 kJ/m^2^ UV or sham-irradiated. (a) Mice treated with AG1478 (right) or vehicle (left) were photographed at 24 h following UV exposure. (b) Skinfold thickness was measured daily following UV. Mean ± standard error is shown. *N* = 10 mice. *Indicates a significant difference compared to the vehicle-treated and sham-irradiated group between 1 and 11 d after irradiation, **significant compared to the vehicle-treated and UV-exposed group between 3 and 9 d after UV, or ***significant compared to the sham-irradiated groups at 2 d and between 4 and 8 d after UV, using two-way ANOVA, where *P* ≤ 0.05. (c) Hematoxylin- and eosin-stained sections revealed increased dermal cellularity in UV-exposed and vehicle treated skin 48 h after UV (200x magnification shown). (d) Neutrophils were counted in at least three 4x microscopic fields in hematoxylin- and eosin-stained sections with the investigator blinded as to the identity of the samples. The mean number of neutrophils per field ± standard error is shown. *N* = 3 mice. (e) Mast cells were counted in 20x microscopic fields in tryptase-stained sections with the investigator blinded as to the identity of the samples. The mean number of tryptase-positive cells per field ± standard error is shown on left and representative images from UV-irradiated skin at the 24 h time point. *N* = 3 mice. **Indicates a significant difference compared to the vehicle-treated control.

To investigate the role of EGFR on the acute inflammatory response, we examined two macroscopic signs of acute UV-induced skin damage, erythema and edema, in mice topically treated with the EGFR inhibitor AG1478 or with the vehicle alone two hours prior to exposure to 10 kJ/m^2^ UVA/B. Within a few hours following a single UV exposure, skin erythema developed in both EGFR inhibitor- and vehicle-treated mice (data not shown). Twenty-four hours after UV, vehicle-treated skin showed severe skin redness (Figure 1(a), left panels). In contrast to vehicle-only treated skin, a single topical application of AG1478 prior to UV irradiation markedly lessened the severity of erythema when mice were examined at the same time point (Figure 1(a), right panels). Similar observations were made in experiments in which the inhibitor was injected intraperitoneally rather than topically applied (data not shown), indicating that the decreased erythema after AG1478 did not result from a sunblocking effect.

UV-associated edema, as measured by skin-fold thickness, was greater in both EGFR inhibitor- and vehicle-treated mouse skin when compared with sham-irradiated skin (Figure 1(b)). Skin-fold thickness in both groups was greatest at 4-5 days after UV exposure. AG1478 application resulted in less than half as much edema at four and five days after irradiation with less edema in inhibitor-treated skin for the duration of the 11 d experiment (Figure 1(b)). Intraperitoneal injection of AG1478 prior to UV irradiation similarly suppressed the increase in skin-fold thickness (data not shown). Thus, EGFR activation correlates positively with the macroscopic signs of skin edema and erythema following UV exposure. 

### 3.2. Inhibition of EGFR Decreases UV-Induced Neutrophil and Mast Cell Infiltration

To further elucidate the role of EGFR in the inflammatory response to UV, the skin was examined histologically for signs of inflammation after UV irradiation. Hematoxylin- and eosin-stained skin sections from vehicle treated and UV-exposed skin revealed increased dermal cellularity, consistent with infiltration of inflammatory cells, when compared to the sham-irradiated control (Figure 1(c), top). In contrast, inhibition of EGFR suppressed this response (Figure 1(c), bottom). Inhibition of EGFR resulted in less damage to the epidermis and reduced dermal cellularity, consistent with decreased inflammation in the skin (Figure 1(c), bottom). 

To further investigate the effect of inhibition of EGFR on inflammation, neutrophils and mast cells were examined. Neutrophils play roles in acute inflammation following UV exposure and contribute to angiogenesis following UV through the release of human leukocyte elastase that degrades dermal elastin [25]. Neutrophil numbers were quantified in hematoxylin- and eosin-stained skin sections from vehicle- and inhibitor-treated UV- and sham-irradiated skin. While the baseline number of neutrophils in sham-irradiated controls was not significantly different from that of inhibitor-treated skin, a single UV exposure increased neutrophil skin infiltration to a greater extent in the vehicle-treated mice (Figure 1(d)). A significant increase in neutrophil number was detected in vehicle-treated skin as early as 16 h after UV-irradiation (Figure 1(d)). The number of neutrophils continued to increase in vehicle treated skin to a maximum of 23.4 neutrophils per microscopic field at 48 h (Figures 1(c)-1(d)). In contrast, in inhibitor-treated skin neutrophils did not increase until 24 h after UV and peaked at only 5.3 neutrophils per microscopic field at this time, less than half of the number in the vehicle-treated group. By 48 h neutrophil number declined to 1.6 neutrophils per field in the inhibitor treated mice, less than 10% of that in the vehicle treated skin (Figures 1(c)-1(d)).

In addition to neutrophils, mast cells participate in the UV-induced skin inflammatory response [26–28]. For example, mast cells are believed to contribute to UV-induced sun damage through the release of potent pro-inflammatory mediators, including histamine, leukotrienes, and tumor necrosis factor-*α* [28]. The effect of EGFR inhibition of mast cells was examined in tryptase-stained skin sections. Sham-irradiated skin pretreated with AG1478 showed a similar number of mast cells when compared to vehicle-treated skin (Figure 1(e)). UV exposure increased mast cell number in vehicle-treated skin by 18 h, with an even greater increase at 24 and 48 h after UV (Figure 1(e)). In contrast, mast cell numbers in inhibitor treated skin did not significantly increase in the first 24 h after UV (Figure 1(e)). At 24 h after UV, the number of mast cells in inhibitor-treated skin was less than half that seen in the sham-irradiated skin (Figure 1(e)). By 48 h after UV, inhibitor-treated skin did have increased numbers of mast cells although they were still fewer than those in the vehicle-treated skin. These data indicate that UV-dependent EGFR activation regulates mast cell and neutrophil infiltration within the skin. 

### 3.3. EGFR Activation in Response to UV Regulates the Expression of IL-1*α*, TNF-*α*, and KC

**Figure 2:**
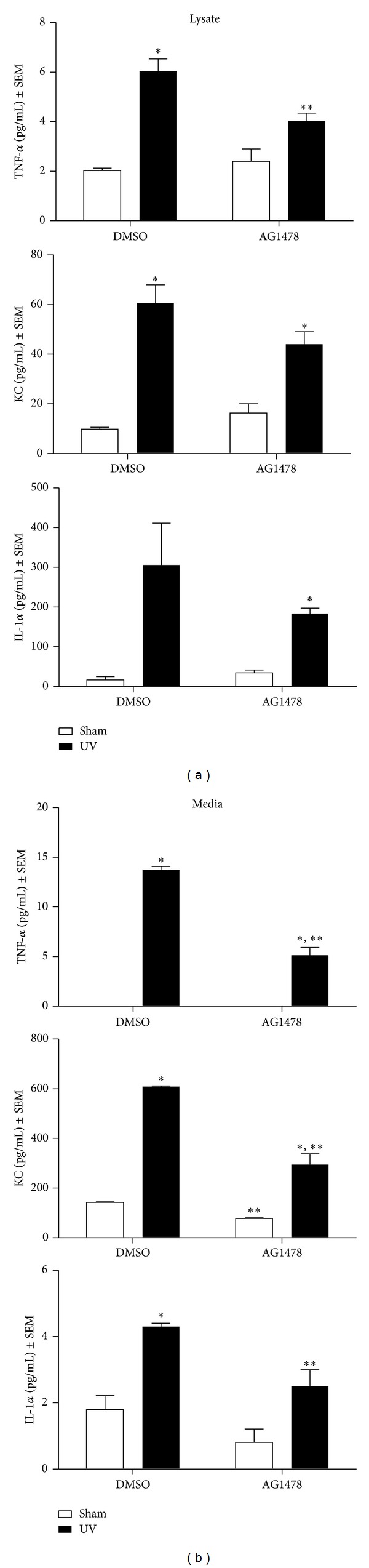
EGFR inhibition reduces TNF-*α*, KC, and IL-1*α* in keratinocytes following UV exposure. Subconfluent keratinocytes were treated with 1 *μ*M AG1478 or the vehicle DMSO. Cells were exposed to 600 J/m^2^ UV and cell lysate (left panel) and media (right panel) were prepared at 16 h for cytokine analysis using Luminex technology. Data from at least two different experiments, with at least three dishes per group, are presented as mean ± standard error. *Mean is significantly different from the corresponding sham-irradiated control or **significantly different from the corresponding vehicle-treated group, using a Student's *t*-test, where *P* ≤ 0.05.

To determine whether pharmacological inhibition of EGFR would affect cytokine production following UV irradiation, cultured keratinocytes were pretreated with the EGFR inhibitor AG1478 and UV exposed. Since EGFR phosphorylation was detected in keratinocytes 5 minutes following exposure to a range of UV exposures from 100 to 600 J/m^2^ of UV (data not shown), cells were irradiated with 600 J/m^2^ UV and cytokine levels were measured in both cell lysate and in media 16 h following UV exposure or sham-irradiation (Figure 2). KC (the murine IL-8 homolog) and IL-1*α* were detectable in both lysate and media of sham irradiated cells while TNF-*α* was only detectable in the cell lysate. No significant differences between sham-irradiated and vehicle or inhibitor-treated keratinocytes were detected (Figure 2, white bars). As expected, UV exposure enhanced production of TNF-*α*, KC, and IL-1*α* cytokines in keratinocytes and in keratinocyte-conditioned media (Figure 2, black bars compared to white bars). Cells pretreated with the EGFR inhibitor had reduced levels of TNF-*α*, KC, and IL-1*α* in both conditioned media and of TNF-*α* in cell lysates 16 h following UV exposure when compared with DMSO-treated and UV-exposed keratinocytes (Figure 2). 

**Figure 3:**
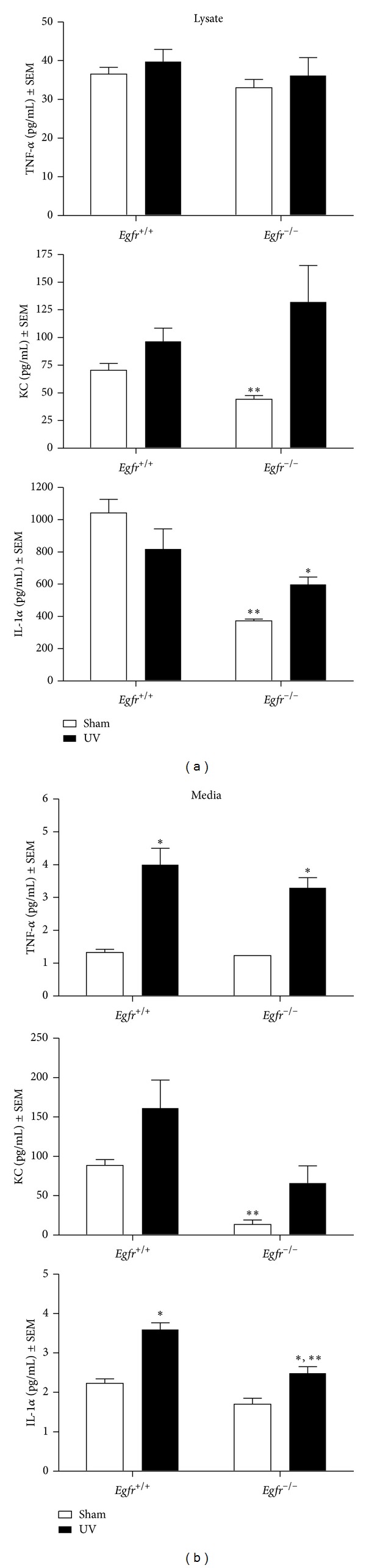
Genetic deletion of *Egfr* reduces TNF-*α*, KC, and IL-1*α* in keratinocytes following UV exposure. Subconfluent *Egfr-*null and wild-type keratinocytes were exposed to 600 J/m^2^ UV irradiation and cell lysate (left panel) and media (right panel) were harvested for cytokine analysis at 16 h using a Luminex instrument. *N* ≥ 4 dishes. *Mean is significantly different from the corresponding sham-irradiated control or **significantly different from the corresponding wild-type group, using a Student's *t*-test, where *P* ≤ 0.05.

To verify the involvement of EGFR in cytokine expression following UV irradiation, *Egfr*-null and wild-type keratinocytes were exposed to 600 J/m^2^ or sham-irradiated and the levels of TNF-*α*, KC, and IL-1*α* were measured in cell lysate and in media 16 h later (Figure 3). Cytokine levels tended to be higher in the controls of this experiment than in the inhibitor experiment, suggesting a suppression of cytokine levels by the vehicle DMSO (Figure 3 compared to Figure 2). Genetic ablation of *Egfr* resulted in a trend toward decreased baseline levels of the KC and IL-1*α* when compared to *Egfr* wild-type cells in both lysate and medium (Figure 3, white bars). Following UV exposure, increased levels of TNF-*α*, KC, and IL-1*α* were measured in wild-type medium, and of TNF-*α* and IL-1*α* in *Egfr* ^−/−^ medium (Figure 3). The magnitude of the increase in TNF-*α*, IL-1*α*, and KC was greater in the *Egfr *wild-type conditioned medium. KC was the only cytokine increased in wild-type lysate, while both KC and IL-1*α* were increased in *Egfr-*null lysate. Taken together, our results suggest that EGFR signaling contributes to increased TNF-*α*, KC, and IL-1*α* levels in sham- and UV-irradiated keratinocytes. 

### 3.4. COX-2 Expression in Keratinocytes Is Regulated by EGFR

**Figure 4:**
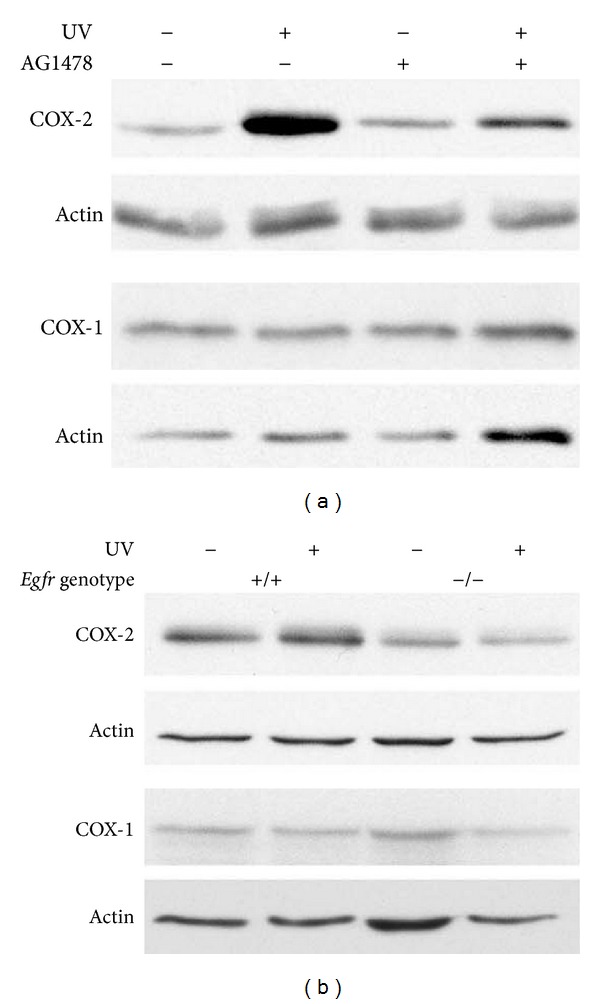
EGFR increases COX-2 levels in keratinocytes following UV exposure. (a) Subconfluent keratinocytes were treated with 1 *μ*M AG1478 or vehicle 2 h prior to 200 J/m^2^ UV irradiation or sham-irradiation. (b) Subconfluent *Egfr*-null and wild-type keratinocytes were UV-exposed (200 J/m^2^) or sham-irradiated. (a)-(b) Sixteen hours after irradiation, protein lysate was prepared. Samples were immunoblotted with the indicated antibodies. Data are representative of three experiments performed with similar results.

UV exposure induces epidermal expression of COX-2, an important enzyme that regulates cytokine production and inflammation [8, 21, 22]. In addition, topical inhibition of COX-2 effectively inhibits UVB-mediated inflammation [29]. To determine whether EGFR contributed to the UV-induced increase in cytokine levels through a mechanism involving COX-2, we examined levels of COX-2 following UV exposure in keratinocytes treated with AG1478 or the vehicle alone and in *Egfr*-null and wild-type keratinocytes. COX-2 protein was increased more than threefold by 16 h following UV exposure of vehicle-treated keratinocytes (Figure 4(a)). Inhibition of EGFR had no effect on COX-2 in sham-irradiated cells (Figure 4(a)). However, pretreatment with the EGFR inhibitor largely prevented the UV-induced increase in COX-2 (Figure 4(a)). As expected, COX-1, the constitutive form, was not increased by UV and inhibition of EGFR did not alter COX-1 levels (Figure 4(a)). 

To determine whether genetic ablation of *Egfr *would produce similar effects on COX-2,* Egfr-*null and wild-type cells were similarly exposed and COX proteins assessed. Baseline levels of COX-2 expression were lower in *Egfr*-null keratinocytes in comparison with wild-type control cells (Figure 4(b)). UV exposure enhanced COX-2 expression only slightly in wild type keratinocytes and not at all in *Egfr*-null keratinocytes (Figure 4(b)). Levels of COX-1 were not affected by deletion of *Egfr* or UV irradiation. Thus, although slightly different results were obtained using the two models for blockade of EGFR signaling, both sets of experiments are consistent with EGFR upregulation of COX-2 after UV exposure.

### 3.5. p38 Kinase Activation in Keratinocytes Is Dependent on UV-Induced EGFR Activation

**Figure 5:**
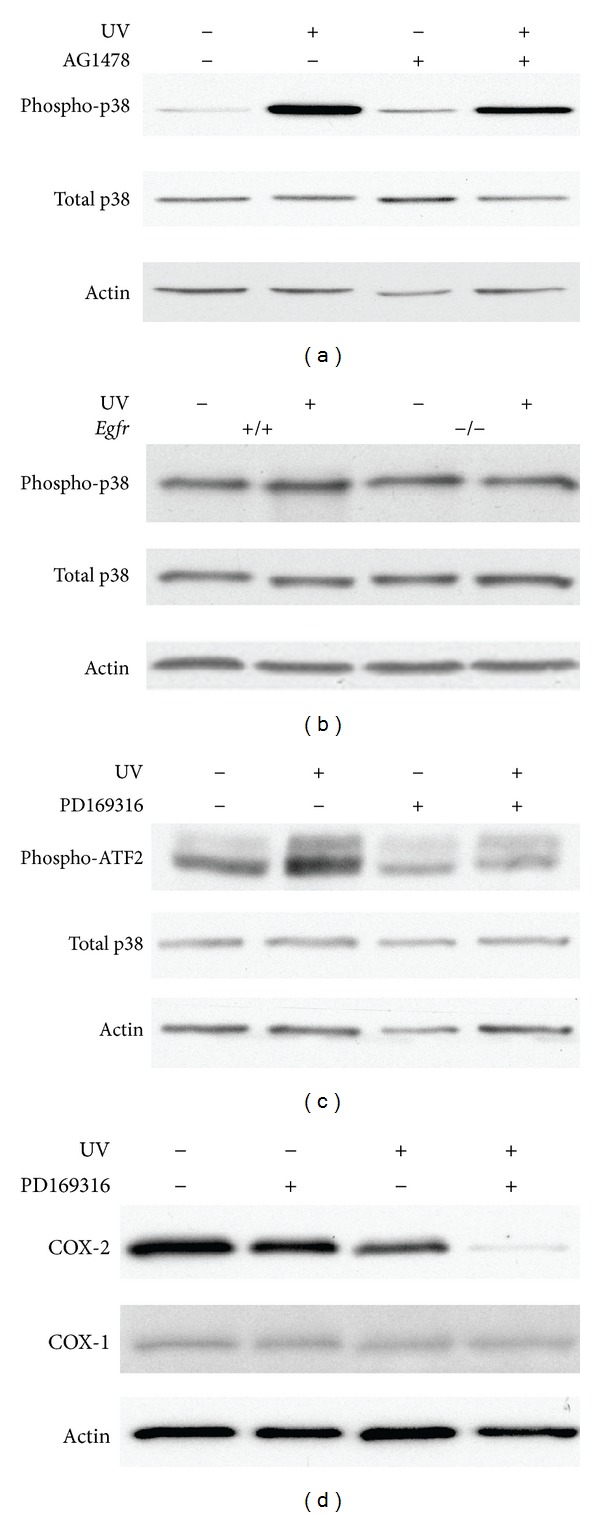
Inhibition of p38 kinase reduces COX-2 in UV-irradiated keratinocytes. Subconfluent wild-type keratinocytes (a)–(d) or *Egfr-*null keratinocytes (b) were treated with AG1478 (a), PD169316 (c), (d), or vehicle alone (a), (c), (d), followed by exposure to 200 J/m^2^ or sham irradiation. Fifteen minutes (a)–(c) or 16 h later (d), protein lysate was prepared. Samples were immunoblotted with the indicated antibodies. Data are representative of three experiments performed with similar results.

Numerous studies have shown that p38 kinase is activated by UV in both human keratinocyte cell lines [21] and in mouse skin [18] Topical inhibition of p38 kinase effectively inhibits UVB-mediated inflammation [18, 30]. In addition, EGFR regulates *in vivo* levels of phosphorylated p38 kinase following UV exposure [3]. To explore the effects of EGFR inhibition on the activation of p38 kinase as a mechanism for COX-2 regulation, wild-type keratinocytes were treated with AG1478 prior to UV exposure or sham irradiation. p38 kinase activity was assessed indirectly by examining p38 kinase phosphorylation on immunoblot. Fifteen minutes following UV exposure, levels of the phosphorylated, active form of p38 kinase were increased in vehicle-treated and UV-exposed cells when compared to sham-irradiated controls (Figure 5(a)). Inhibition of EGFR reduced the UV-stimulated phosphorylation of p38 kinase (Figure 5(a)). In the parallel experiment using *Egfr* wild-type and null keratinocytes, the increase in p38 kinase activity in response to UV was less striking in the control genotype (Figure 5(b)), when compared to the vehicle-treated keratinocytes of Figure 5(a). However, genetic deletion of *Egfr* further suppressed p38 kinase phosphorylation in response to UV (Figure 5(b)). Thus, activation of p38 kinase by UV is partially dependent on EGFR signaling.

### 3.6. Inhibition of p38 Kinase Reduced EGFR-Induced COX-2 Expression in Response to UV Irradiation

To investigate whether p38 kinase-dependent signaling is required for UV-induced expression of COX-2, we used the p38 kinase inhibitor PD169316. UV exposure activated p38 kinase as reflected by the increased phosphorylation of the p38 kinase substrate activating transcription factor-2 (ATF2) [30] (Figure 5(c)). *Egfr *wild-type keratinocytes were treated with 5 *μ*M inhibitor for 1 h before exposure to UV, with continuing incubation in the presence of the inhibitor. Inhibition of p38 kinase reduced the activation of p38 as detected by reduced phosphorylated ATF2 when compared to sham-irradiated levels at 15 minutes following UV (Figure 5(c)). In addition, inhibition of p38 kinase prior to UV exposure reduced COX-2 in *Egfr* wild-type cells at 16 h (Figure 5(d)), demonstrating that p38 kinase activity is necessary for full expression of COX-2 after UV irradiation.

## 4. Discussion

In this study, we examined the direct involvement of EGFR signaling in mediating skin inflammation upon UV exposure. We found that inhibition of EGFR suppressed UV-induced edema and erythema in mouse skin. Similarly, neutrophil and mast cell infiltration of the skin following UV exposure were also suppressed with blockade of EGFR. Using cell culture models, we further examined cytokine levels in EGFR inhibitor-treated and *Egfr-*null keratinocytes. Some differences in the response to UV were detected between the inhibitor and genetic models, suggesting an effect of the vehicle DMSO. Taken together, however, these experiments demonstrated that decreased levels of TNF-*α*, KC (mouse homolog of IL-8), IL-1*α*, and COX-2 in response to UV resulted from abrogation of EGFR in mouse keratinocytes. Inhibition of the EGFR-activated p38 kinase similarly suppressed COX-2 levels in UV-irradiated keratinocytes, suggesting EGFR activation of p38 kinase as a potential mechanism for the increases in COX-2 and cytokines associated with UV-induced inflammation. 

The inflammatory response to UV is likely the result of both EGFR-dependent and EGFR-independent signaling pathways. MAPK transduction pathways are involved in modulating cytokine production in mouse skin inflammatory response to chemical irritants [31, 32]. In particular, p38 kinase is activated by phosphorylation in keratinocytes *in vitro* [21] and *in vivo* [33] in response to UV. Therefore, we investigated whether EGFR regulates p38 kinase activation in response to UV. Our analyses revealed that p38 kinase is activated in response to UV in manner partially dependent on EGFR. In addition, our data show that COX-2 expression is largely dependent on EGFR-mediated p38 kinase activation. Previous studies using murine models have demonstrated that topical inhibition of COX-2 after UV exposure inhibited inflammation [29, 34], presumably through decreased production of PGE2 and vascular endothelial growth factor (VEGF), reducing erythema and edema [8, 27, 29, 35, 36]. Interestingly, COX-2 is highly expressed in murine and human nonmelanoma UV-induced skin tumors, while inhibition of COX-2 appears to decrease such tumor formation [37–39]. Furthermore, since some authors have reported that COX-2-derived PGE2 synthesis is a key event of skin tumor promotion in response to UV [34], COX-2 suppression resulting from EGFR inhibition is worthy of further investigation. 

In addition to the EGFR-dependent up-regulation of COX-2 through p38 kinase documented here, additional EGFR-dependent mechanisms regulating inflammation have been documented. For example, activated MEK1-ERK signaling contributes to increased IL-1*α* production [31] and TNF-*α* expression [32] in keratinocytes. Similarly, EGFR signaling through MEK1/2 and p38 kinase synergizes with IL-1*α* in the skin innate immune response by enhancing the production of antibacterial peptides in normal skin and chronic inflammatory diseases like psoriasis [40]. In addition, an immunomodulatory role of EGFR was suggested by the increased expression of granulocyte/macrophage colony- stimulating factor (GM-CSF), a pro-inflammatory cytokine in mouse and human skin [31, 32, 41], although this finding has not been investigated in the context of UV-induced skin inflammation.

Skin infiltration with neutrophils and mast cells is a hallmark of various skin inflammatory disorders and in that of UV-induced inflammation [27, 42, 43]. Our findings revealed that abrogation of EGFR signaling suppressed the increase in dermal neutrophils and mast cells following UV exposure. Since these recruited inflammatory cells release various cytokines and enzymes that enhance vascular permeability, suppression of such inflammatory cell infiltration would be expected to lessen the degree of inflammation. For example, Meyer-Hoffert et al. have found that EGFR-neutralizing antibodies abolish the proliferative effect of human leukocyte elastase produced by neutrophils in human keratinocytes [43]. Moreover, reduction of infiltrating mast cells with their attendant degranulation in response to UV appears to alleviate inflammation, possibly through reduced release of mast cells specific products, such as histamine, TNF-*α*, and the delayed phase of PGE2 synthesis mediated by COX-2 [28, 42]. In addition, mast cells are required for the melanocyte activation induced by endothelin-1 and, hence, the protective tanning response to UV [27]. 

In addition to the suppression of acute inflammation after UV exposure documented here, abrogation of EGFR function is also well known to lead to cutaneous inflammation, more specifically to folliculitis, in cancer patients undergoing longer-term treatment with EGFR inhibitors [44]. Thus, the role of EGFR in cutaneous inflammation is certainly complex. Because of the apparent multifaceted functions of EGFR in regulating cutaneous inflammation, further investigation into the role of EGFR-dependent pro- and anti-inflammatory effects is warranted. 

## 5. Conclusions

Based on our data, we conclude that EGFR signaling contributed to UV-induced inflammation, potentially through multiple mechanisms. EGFR activation increased mast cell and neutrophil numbers in the skin, edema, and erythema, responses that may occur through both EGFR/p38 kinase/COX-2-dependent and independent mechanisms. Since we previously found that ErbB2 mediates skin inflammation in response to UV [45], examining the effects of combination therapies which target major activated receptor tyrosine kinases in skin following UV irradiation will prove interesting.
